# The treatment efficacy of cortical bone trajectory (CBT) pedicle screws for lumbar degenerative disease in the Chinese Han population

**DOI:** 10.3389/fsurg.2022.421815

**Published:** 2022-10-31

**Authors:** Jinhui Wu, Tao Lin, Heng Jiang, Jun Ma, Ke Zhang, Jianquan Zhao, Xuhui Zhou, Ce Wang

**Affiliations:** Orthopaedic Surgery, Shanghai Changzheng Hospital, Second Military Medical University, Shanghai, China

**Keywords:** CBT, degenerative lumbar disease, outcome, HRQOL (health-related quality-of-life), morphometric measurement

## Abstract

**Purpose:**

To provide reference data on CBT pedicle screws from CT measurements of L1 to L5 in the Chinese Han population and to assess the treatment efficacy of CBT pedicle screws in cases of lumbar degenerative disease.

**Methods:**

In total, 100 patients were identified from the CT database for CBT morphometric measurement of the lumbar spine. According to sex and age, patients were divided into four groups. The diameter, length, and angle of the vertebral pedicle and trajectory were measured. Then, a total of 36 patients with lumbar degenerative disease were included in this study for clinical and radiographic evaluation. Demographic characteristics, health-related quality of life (HRQOL), and extent of intervertebral disc herniation and spondylolisthesis were evaluated.

**Results:**

The mean diameter and the mean length varied from L1 to L5 in Groups I to IV. The lateral angles ranging from L1 to L5 were 8.9 to 9.2°, 8.7 to 12.2°, 8.7 to 11.2°, and 9.2 to 10.1° in Groups I to IV, respectively. The cephalad angles from L1 to L5 were 23.5 to 28.6°, 24.7 to 26.6°, 25.0 to 28.2°, and 24.7 to 27.9° in Groups I to IV, respectively. In the case series, all patients’ neurological function and HRQOL were significantly improved at the final follow-up (*p* < 0.0001), and 75% of patients achieved satisfaction.

**Conclusions:**

The morphology of the lumbar vertebral pedicle varied from L1 to L5, and the trajectory was feasible and safe. CBT pedicle screws were effective in treating lumbar degenerative diseases and benefited the patients.

## Introduction

Pedicle screws are accepted worldwide for lumbar fixation, and various benefits have been observed since Roy-Camille raised the concept of pedicle screws in 1970 (1). However, pedicle screw loosening is a challenging postoperative complication, especially in people with poor bone quality, such as osteoporotic bone (2–4). An estimated 44 million Americans suffered from osteoporosis in 2000 (5); meanwhile, approximately 6.97% of Chinese people faced the same problems, and the severity was positively correlated with age according to a survey in 2000 (6), which indicated a high risk of screw loosening. Several methods have been proposed to increase the pullout strength and structural stability to avoid this problem. The modification of screw design is a common method, including the alteration of thread pitch and shape, and expandable screws have also been considered. However, these methods have controversial efficacy and might have risks of causing pedicle fracture and neurologic damage (7, 8). Another generally accepted method is to implant hard material into the vertebrae to reinforce the structure, including bone cement and polymethylmethacrylate (PMMA), which several biomechanical and clinical experiments have proven to be effective. However, the complications of high exothermic polymerizing temperature, toxicity, and poor fatigue performance still cannot be ignored (9–11).

Recently, Santoni et al. (12) proposed an applicable method to improve the stability of lumbar fixation called the cortical bone trajectory (CBT) pedicle screw. This is a novel trajectory method that changes the pedicle screw insertion from the traditional transpedicular path through the anatomic axis of the pedicle to a new trajectory that follows a caudocephalad path sagittally and a lateral path in the transverse plane (13, 14). Several researchers have reported the efficacy of this method biomechanically and clinically. Snyder et al. (15) investigated 79 patients who had a degenerative lumbar disease and received fixation of CBT pedicle screws, and 91.1% of them achieved satisfactory solid fusion. Koshi et al. (16) made a comparison of conventional pedicle screws and CBT pedicle screws in curing degenerative lumbar spondylolisthesis and found reliable stability of CBT pedicle screws. Masaki et al. (14) performed a biomechanical experiment and found that the maximum pullout strength was significantly greater for CBT pedicle screws than for traditional trajectory pedicle screws. Cheng et al. (17) and Perez et al. (18) mentioned similar results. However, the clinical outcomes and trajectory data may be significantly varied according to different ethnicities. To the best of our knowledge, few previous studies have focused on the efficacy of CBT pedicle screws combined with relative trajectory data from the Chinese Han population, which inspired us to study this intersection.

In our study, we aimed to achieve the following: (1) evaluate the efficacy of degenerative lumbar disease treatment through the fixation of CBT pedicle screws in patients and (2) provide reliable morphometric data of lumbar vertebrae and trajectories based on computed tomography (CT, from L1 to L5) in a Chinese Han population.

## Materials and methods

### Patients

A retrospective study was performed involving 36 patients who received lumbar CBT pedicle screws in our department. The search of the CT database returned 100 patients with lumbar CBT morphometric measurements from January 2014 to December 2016 in Shanghai Changzheng Hospital. All the enrolled patients were from the Chinese Han population. The research project was approved by the ethics committee of Shanghai Changzheng Hospital, Shanghai, China. We gained consent from all participants. All procedures were performed under the Declaration of Helsinki and followed relevant policies in China.

Patients who met criteria (1) and (2) combined with (3) or (4) were enrolled. (1) Patients with a follow-up of at least 2 years had complete preoperative and postoperative lumbar anteroposterior and lateral x-ray, computed tomography (CT), and magnetic resonance imaging (MRI); (2) Patients presented with severe low back pain with or without numbness of the lower limbs and sciatica; (3) Grade I to IV lumbar intervertebral disc herniation at L1 to L5 (Pfirrmann criteria) was present; (4) Lumbar ligament ossification and spondylolisthesis at L1 to L5 (grade 0 to IV, Meyerding criteria) were present. Patients who had comorbidities or habits that would influence bone healing, such as smoking, diabetes, multiple myeloma, ankylosing spondylitis or tumors, congenital malformations, or neuromuscular or traumatic diseases that caused defects in the vertebrae were excluded from this study.

The indications of fusion surgery included lumbar spondylolisthesis, lumbar instability, lumbar spinal nerve canal stenosis, lumbar discogenic pain, and recurrent lumbar disc herniation.

### Clinical evaluation

Age, sex, symptoms, body mass index (BMI), estimated blood loss (EBL), operation room time (ORT), follow-up time, and preoperative complications were evaluated. The lumbar Japanese Orthopedic Association (JOA) score was calculated to assess neurologic conditions before and after surgery.

We adopted the Oswestry Disability Index (ODI) to assess the health related quality of life (HRQOL) of the patients before and after surgery. To decrease errors, the concept of minimum clinically important difference (MCID) was introduced, which is the smallest amount of improvement that is clinically relevant to the individual patient for various outcome measures. The MCID marks the absolute minimum change that can be considered a success and serves as a starting point for an analysis of actual, patient improvement (19, 20). In our study, the MCID of ODI is −15 from the literature (21), and a change in ODI score >+1 MCID was considered satisfactory and other conditions were considered unsatisfactory (Equation 1).

(1) $$\mathrm{OD}I_{\mathrm{Diff}}=\frac{\mathrm{OD}I_{\mathrm{postop}}-\mathrm{OD}I_{\mathrm{preop}}}{\mathrm{OD}I_{\mathrm{MCID}}}$$

Method to calculate the changes of ODI score. ODI, oswestry disability index; Postop, postoperative; Preop, preoperative; MCID, minimum clinically important difference. ODI_Diff_ > +1MCID is considered as satisfaction.

### Morphometric measurements of lumbar CBT

A total of 100 patients were found by searching the CT database for morphometric measurements. The lumbar spines of these patients were normal and not affected by a tumor, an infection, or a fracture. These patients were divided into four groups: male patients ≤45 years old in Group I, male patients ≥60 years old in Group II, female patients ≤45 years old in Group III, and female patients ≥60 years old in Group IV. Every group included 25 patients.

**Figure 1:**
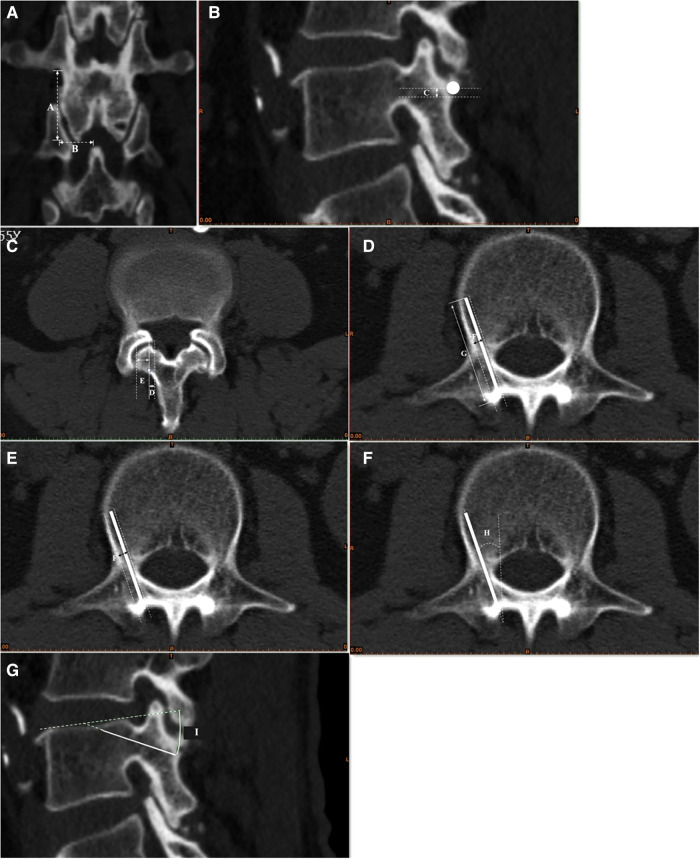
Morphometric measurement of the trajectory of the CBT screw. (**A**) The height (mm) of the lumbar pedicle (A), the width (mm) of the lumbar pedicle (B); (**B**) the distance (mm) of the inferior margin of the pedicle to the starting point (C); (**C**) the distance (mm) of the medial margin of the pedicle to the starting point (D), the distance (mm) from the starting point to the lateral margin of the pars interarticularis (E); (**D**) the maximum diameter (mm) in the transverse plane (F); (**E**) the maximum length (mm) in the sagittal plane (G); (**F**): the lateral angle (°) (H); (**G**): the cephalad angle (°) (I).

Several CT measurement data were collected as follows: the height (mm) of the lumbar pedicle (A), the width (mm) of the lumbar pedicle (B), the distance (mm) of the inferior margin of the pedicle to the starting point (C), the distance (mm) of the medial margin of the pedicle to the starting point (D), the distance (mm) from the starting point to the lateral margin of the pars interarticularis (E), the maximum diameter (mm) in the transverse plane (F), the maximum length (mm) in the sagittal plane (G), the lateral angle (°) (H), and the cephalad angle (°) (I). All the methods of measurement are shown in Figure 1.

### Radiographic evaluation

The grades of intervertebral disc herniation and spondylolisthesis were evaluated according to MRI and x-ray, respectively. Screw loosening was also assessed by CT. A radiolucency >1 mm surrounding a screw, regardless of the length of lucency along the screw axis, was defined as a radiolucent zone, which indicated pedicle screw loosening (22, 23).

### Statistical analysis

Statistical analysis was performed using SPSS 19.0 software (SPSS Inc., Chicago, IL). Differences were compared by Student’s t test and Wilcoxon signed-rank test. A two-tailed *p* value < 0.05 was considered to be statistically significant.

## Results

For morphometric measurement groups, the average ages of Groups I, II, III, and IV were 32.2 ± 5.3, 63.4 ± 2.8, 35.9 ± 8.4, and 65.0 ± 4.0 years old, respectively. CT images were reconstructed by three-dimensional reconstruction software (Philips Intellispace Portal) to measure the trajectory data. We provided specific data separately according to each group and level of lumbar vertebrae (Table 1). The height (mm) of the lumbar pedicle (A) tended to decrease slightly from L1 to L4 except for a slight increase at L5 in each group (L1 to L4: 17.3 ± 2.1 to 16.3 ± 1.8 mm and L5: 18.3 ± 3.1 mm in Group I, L1 to L4: 18.1 ± 1.3 to 16.1 ± 1.9 mm and L5: 13.9 ± 1.5 mm in Group II, L1 to L4: 16.2 ± 1.6 to 14.8 ± 1.4 mm and L5: 18.0 ± 2.3 mm in Group III, and L1 to L5: 16.9 ± 1.2 to 15.6 ± 0.8 mm and L5: 17.9 ± 1.9 mm in Group IV). The width (mm) of the lumbar pedicle (B) gradually increased from L1 to L5 in all groups (10.4 ± 2.0 to 28.2 ± 5.6 mm in Group I, 9.7 ± 1.0 to 30.0 ± 4.7 mm in Group II, 7.7 ± 1.7 to 27.3 ± 4.9 mm in Group III, and 8.9 ± 1.6 to 23.5 ± 4.3 mm in Group IV). The distance (mm) of the inferior margin of the pedicle to the starting point (C) showed an increasing tendency from L1 to L5 in all groups (1.3 ± 1.1 to 3.7 ± 2.0 mm in Group I, 1.5 ± 1.4 to 2.9 ± 1.2 mm in Group II, 1.5 ± 1.2 mm to 5.3 ± 2.8 mm in Group III, and 1.5 ± 0.7 to 4.6 ± 2.1 mm in Group IV). The distance (mm) of the medial margin of the pedicle to the starting point (D), the distance (mm) from the starting point to the lateral margin of the pars interarticularis (E), the maximum diameter (mm) in the transverse plane (F), and the maximum length (mm) in the transverse plane (G) varied from L1 to L5, and specific data are shown in Table 4. The lateral angle (°) (H) and the cephalad angle (°) (I) were also measured in our study, and there were no significant differences between each level (*p* > 0.05).

**Table 1:** Morphometric measurements of CBT pedicle screws (mean ± SD).

	Group I					Group II				
	L1	L2	L3	L4	L5	L1	L2	L3	L4	L5
A (mm)	17.3 ± 2.1	17.3 ± 1.6	16.9 ± 1.7	16.3 ± 1.8	14.3 ± 3.1	18.1 ± 1.3	17.7 ± 1.6	16.5 ± 1.6	16.1 ± 1.9	13.9 ± 1.5
B (mm)	10.4 ± 2.0	10.7 ± 2.2	13.0 ± 2.2	16.7 ± 3.4	28.2 ± 5.6	9.7 ± 1.0	10.3 ± 1.4	14.1 ± 1.9	18.8 ± 2.5	30.0 ± 4.7
C (mm)	1.3 ± 1.1	1.4 ± 1.0	1.2 ± 1.0	1.4 ± 1.2	3.7 ± 2.0	1.5 ± 1.4	1.6 ± 0.8	1.2 ± 0.6	1.3 ± 0.6	2.9 ± 1.2
D (mm)	2.0 ± 1.1	1.8 ± 1.3	2.3 ± 1.5	2.7 ± 1.7	3.7 ± 1.8	1.6 ± 0.8	2.2 ± 1.2	2.2 ± 1.0	2.4 ± 0.9	5.8 ± 1.7
E (mm)	1.8 ± 0.7	2.7 ± 0.8	3.5 ± 1.1	4.7 ± 1.3	5.9 ± 1.8	1.9 ± 0.8	2.5 ± 0.9	3.2 ± 0.9	4.3 ± 0.9	10.1 ± 1.7
F (mm)	7.7 ± 1.4	7.4 ± 1.4	7.9 ± 1.5	8.6 ± 1.7	9.2 ± 1.4	7.7 ± 1.2	7.9 ± 1.3	8.6 ± 1.0	9.3 ± 1.4	11.6 ± 2.8
G (mm)	35.5 ± 2.9	37.5 ± 4.4	38.9 ± 4.4	39.4 ± 4.0	39.0 ± 3.9	35.7 ± 4.0	38.6 ± 3.0	40.1 ± 2.5	40.1 ± 2.4	39.9 ± 2.7
H (°)	9.0 ± 3.8	9.0 ± 3.0	9.2 ± 3.5	9.0 ± 3.1	8.9 ± 2.9	8.7 ± 1.2	9.8 ± 2.3	12.2 ± 2.5	10.9 ± 2.2	11.6 ± 2.8
I (°)	28.1 ± 4.1	26.5 ± 6.1	23.5 ± 4.6	27.9 ± 5.5	28.6 ± 3.7	26.1 ± 4.1	26.4 ± 4.1	24.7 ± 4.7	25.9 ± 4.3	26.6 ± 4.1
A (mm)	16.2 ± 1.6	15.9 ± 1.5	15.4 ± 1.4	14.8 ± 1.4	13.0 ± 2.3	16.9 ± 1.2	16.0 ± 1.0	16.1 ± 0.9	15.6 ± 0.8	13.9 ± 1.9
B (mm)	7.7 ± 1.7	8.3 ± 2.2	11.1 ± 2.2	14.5 ± 2.5	27.3 ± 4.9	8.9 ± 1.6	8.0 ± 1.0	11.0 ± 1.8	14.2 ± 2.1	23.5 ± 4.3
C (mm)	1.5 ± 1.2	1.5 ± 1.0	1.8 ± 1.4	1.4 ± 1.2	5.3 ± 2.8	1.5 ± 0.7	1.4 ± 0.9	1.4 ± 0.7	1.8 ± 0.9	4.6 ± 2.1
D (mm)	1.4 ± 1.1	1.5 ± 0.9	2.8 ± 1.3	2.5 ± 1.4	3.9 ± 1.4	1.8 ± 0.9	1.8 ± 0.8	2.8 ± 1.2	2.5 ± 1.1	3.1 ± 1.6
E (mm)	2.1 ± 0.8	2.7 ± 0.9	3.7 ± 1.5	4.4 ± 1.0	5.4 ± 1.3	2.4 ± 0.7	2.8 ± 0.9	3.2 ± 1.1	3.6 ± 1.1	5.4 ± 1.2
F (mm)	5.8 ± 1.6	5.8 ± 1.3	6.3 ± 1.2	6.8 ± 1.9	7.8 ± 1.6	6.4 ± 1.3	5.8 ± 1.3	6.7 ± 1.1	6.7 ± 1.2	8.1 ± 1.6
G (mm)	35.3 ± 3.4	37.0 ± 4.3	37.1 ± 3.2	35.8 ± 3.9	36.1 ± 3.9	33.4 ± 2.5	34.8 ± 2.9	37.1 ± 3.1	38.4 ± 3.3	39.0 ± 2.5
H (°)	8.8 ± 3.2	8.7 ± 2.6	10.9 ± 2.9	11.2 ± 3.9	10.5 ± 4.3	9.2 ± 2.4	9.6 ± 3.2	9.5 ± 2.6	9.4 ± 3.4	10.1 ± 3.0
I (°)	26.6 ± 5.7	27.3 ± 5.9	25.0 ± 6.6	27.7 ± 3.6	28.2 ± 3.7	25.8 ± 3.4	26.3 ± 3.2	24.7 ± 3.3	27.5 ± 3.2	27.9 ± 3.4

Note: A: the height of the lumbar pedicle; B: the width of the lumbar pedicle; C: the distance of the inferior margin of pedicle to the starting point; D: the distance of the medial margin of pedicle to the starting point; E: the distance from the starting point to the lateral margin of the pars interarticularis; F: the maximum diameter in the transverse plane; G: the maximum length in the sagittal plane; H: the lateral angle; I: and the cephalad angle; Group I: man and age ≤ 45 years old; Group II: man and age ≥ 60 years old; Group III:woman and age ≤ 45 years old; Group IV: woman and age ≥ 60 years old A: the height of the lumbar pedicle; B: the width of the lumbar pedicle; C: the distance of the inferior margin of pedicle to the starting point; D: the distance of the medial margin of pedicle to the starting point; E: the distance from the starting point to the lateral margin of the pars interarticularis; F: the maximum diameter in the transverse plane; G: the maximum length in the sagittal plane; H: the lateral angle; I: and the cephalad angle; Group I: man and age ≤ 45 years old; Group II: man and age ≥ 60 years old; Group III:woman and age ≤ 45 years old; Group IV: woman and age ≥ 60 years old.

**Figure 2:**
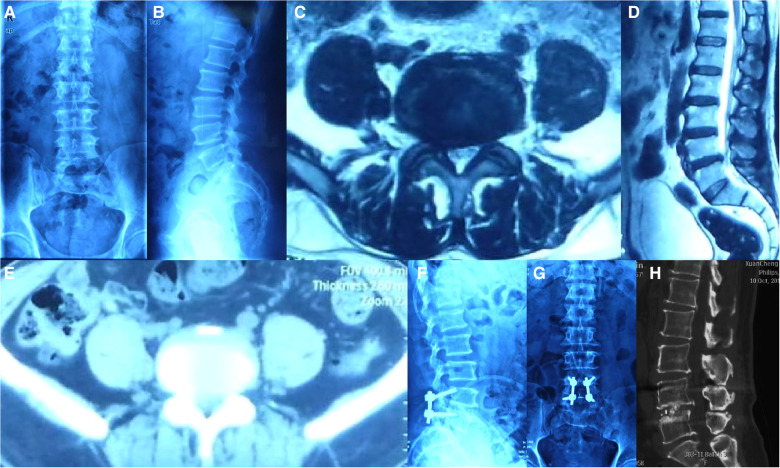
A fifty-two-year-old male patient, presented with low back pain radiating to the left leg for almost 5 years and numbness of both lower limbs for almost 4 years. Physical examination showed that the straight leg raising test of the left leg was positive (60°), decreased muscle power for left tibialis anterior (Manual Muscle Test grade IV), Eaten(−), Spurling(−). MRI revealed a herniation disc at L4/L5 level. Postoperative x-ray showed perfect bone fusion and no internal fixation was a failure. (**A,B**) preoperative anteroposterior x-ray; (**C**) preoperative CT of L4/L5; (**D**) preoperative MRI; (**E,F**) anteroposterior x-ray of 2-years-after-surgery lumbar spine. (**H**) Postoperative sagittal CT.

**Figure 3:**
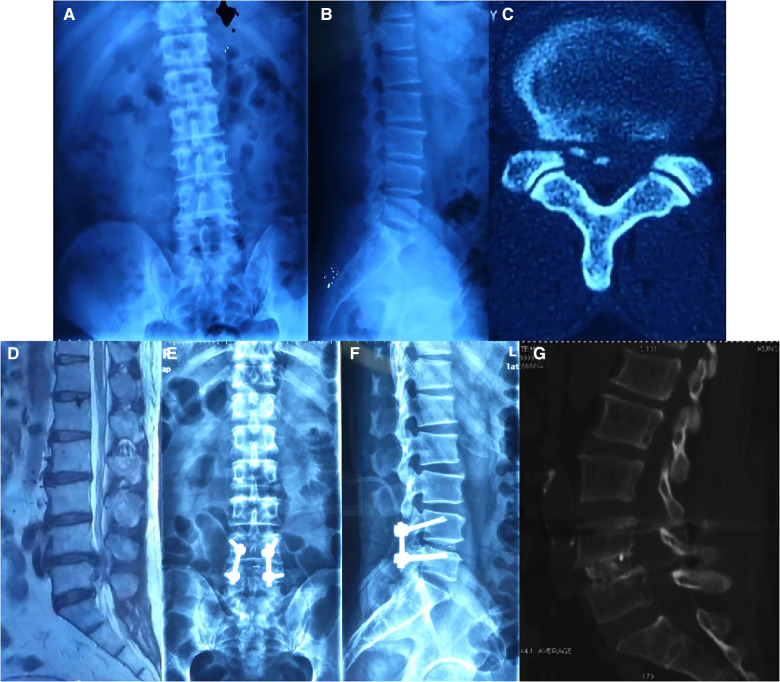
A sixty-one year old male patient, presented with low back pain radiating to both lower limbs, and had numbness of both lower limbs for 4 months. The pain and numbness were exacerbated and intermittent claudication existed for almost 2 months. Decreased muscle power for both iliopsoas, quadriceps femoris, semitendinosus, tibialis anterior, and triceps surae (Manual Muscle Test grade IV). The straight leg raising test of the left leg was positive (60°). Eaten(−), Spurling(−). MRI revealed a herniation disc at L4/L5 level. Postoperative x-ray showed perfect bone fusion and no internal fixation was a failure. (**A,B**) Preoperative anteroposterior x-ray; (**C,D**) preoperative MRI of L4/L5; (**E,F**) anteroposterior and sagittal x-ray of the lumbar spine 2 years after surgery. (**H**) Postoperative sagittal CT.

For the case series, a total of 36 patients who received CBT screws in our department for clinical and radiographic evaluation (Figures 2, 3) were enrolled in our study, and none of them were absent from our follow-up. Liu et al. (6) mentioned that osteoporosis varied by sex and age (people over 60 years old were at high risk of osteoporosis), which might cause differences in trajectory data. For clinical and radiographic evaluation, we separated 36 patients into 2 groups: Group A (age ≥ 60 years old) and Group B2 (age < 60 years old). The average time of follow-up was 2 ± 2.3 years. The average ages were 31.4 ± 7.4 and 66.7 ± 4.9 years old for Group A and Group B2, respectively. Thirty-one patients presented with low back pain; 28 patients presented with lower limb numbness, 23 patients showed myodynamia, and no patients showed paraplegia. The mean EBL, ORT, and BMI showed no significant differences among the four groups (*p* > 0.05). All patients’ JOA scores improved significantly at the final follow-up (*p* < 0.0001). Group B showed a lower JOA score than Group A (8.4 ± 2.2 vs. 11.3 ± 2.8, *p* < 0.05) before surgery (Table 2).

**Table 2:** Demographic characteristics of patients (mean ± SD or *n*).

	Group A	Group B
Sex	Male: 9	Male: 12
	Female: 7	Female:8
*p* value	0.821	
Age (years)	31.4 ± 7.4	66.7 ± 4.9
BMI (kg/m^2^)	21.4 ± 3.9	20.4 ± 3.4
*p* value	0.417	
Symptom (preoperative)		
Low back pain	14	17
Lower limbs numbness	12	16
Myodynamia	10	13
EBL (ml)	288.3 ± 14.5	289.9 ± 10.9
*p* value	0.708	
ORT (h)	3.4 ± 0.6	3.3 ± 0.4
*p* value	0.554	
JOA score		
Preoperative	11.3 ± 2.8	8.4 ± 2.2
Postoperative	25.7 ± 1.4	27.1 ± 1.3
*p* value	<0.0001	<0.0001

Note: Group A: age < 60 years old; Group B: age ≥ 60 years old; JOA, Japanese orthopaedic association; BMI, body mass index; EBL, estimated blood loss; ORT, operating room time.

We used the ODI scale to assess the HRQOL of patients, and significant improvements were observed at the final follow-up (*p* < 0.0001). To decrease errors, the MCID was adopted in our study to compare patient satisfaction between the two groups. Group A showed significant differences compared with Group B (9 vs. 18, OR=0.125, *p* = 0.023) (Table 3).

**Table 3:** Health related quality of life (HRQOL) of patients based on DOI (mean ± SD or *n*).

	Group A	Group B
ODI		
Preoperative	0.53 ± 0.08	0.58 ± 0.07
Postoperative	0.29 ± 0.07	0.27 ± 0.6
*p* value	<0.0001	<0.0001
ODI_Diff_ > +1MCID	9 (56.25%)	18 (90%)
*p* value	0.023	
OR (>+1/≤+1, 95%CI)	0.125 (0.022–0.715)	

Note: Group A: age < 60 years old; Group B: age ≥ 60 years old; ODI, oswestry disability index; OR, odds ratio; CI, confidential incidence.

The preoperative condition of lumbar intervertebral disc herniation in Group B was significantly different from that in Group A (*p* < 0.05). All patients achieved a solid intervertebral fusion of surgical levels at the final follow-up. Thirteen patients had lumbar ligament ossification, and they were all more than 60 years old. Significant differences were observed in spondylolisthesis before and after surgery in each group (*p* < 0.05). We considered grade 0 to I spondylolisthesis as mild grade (mGrade) and grade II to III as severe grade (sGrade). We compared the severity of spondylolisthesis before and after surgery [i.e., Group A versus Group B (preoperative: *p* = 0.023, mGrade: sGrade: OR = 0.200)]. No significant screw loosening was observed in any patient at the final follow-up (Table 4).

**Table 4:** Radiographic assessment (*n*).

	Group A	Group B
Preoperative intervertebral disc herniation		
Grade I	6	2
Grade II	6	3
Grade III	2	10
Grade IV	2	5
*p* value	0.026	
Spondylolisthesis (preoperative, postoperative)		
Grade 0	6, 10	3, 8
Grade I	4, 4	2, 6
Grade II	2, 1	7, 4
Grade III	4, 1	8, 2
OR (95%CI) (preoperative) (sGrade/mGrade)	0.200 (0.048–0.837)	
*p* value (preoperative)	0.023	

Note: Group A: age < 60 years old; Group B: age ≥ 60 years old; OR, odds ratio; CI, confidential incidence; sGrade: spondylolisthesis Grade II to III; mGrade, spondylolisthesis Grade 0 to I spondylolisthesis.

## Discussion

This study evaluated the safety and efficacy of CBT pedicle screws in treating lumbar degenerative diseases. Demographic characteristics were collected; patients were divided into two groups according to age; and no significant differences were observed between groups (*p* > 0.05, Table 1). Neurological function was assessed through the JOA score, and significant improvements were observed at the final follow-up in each group (*p* < 0.0001). The HRQOL of the patients was assessed by the ODI score, and significant improvements were also observed (*p* < 0.0001, Table 2). To decrease errors, MCID was performed to evaluate the real satisfaction of the patients, and ODI_Diff_ > +1 MCID was considered meaningful. Patients of an increased age were more satisfied than younger patients [Group A (9, 56.25%) vs. Group B (18, 90%): OR = 0.125, *p* = 0.023, Table 2]. We also found that elderly patients showed a more severe grade of intervertebral disc herniation and spondylolisthesis (*p* < 0.05), and all patients improved significantly after surgery (*p* < 0.05) (Table 3).

All cases of lumbar spondylolisthesis and intervertebral disc herniation improved, and no further vertebral slippage, screw loosening, or pseudarthrosis were observed in our study, which indicated a superior efficacy in curing lumbar degenerative diseases. Goldstein et al. (24), Orribo et al. (18), and Chin et al. (25) shared similar opinions to ours. Traditional pedicle screws have been adopted effectively in curing lumbar degenerative diseases for decades, and several, previous studies have compared the efficacy between traditional pedicle screws and CBT screws. Lee et al. (26) conducted a cohort study of comparison and found that both screws showed similar fusion rates and stability. Ninomiya et al. (16) believed that the reduction ratio of lumbar vertebral slippage was similar in both screws, and a similar good initial fixation was also observed. Thus, CBT was an alternative to traditional pedicle screws based on the radiological evaluation. Some biochemical studies suggested that increasing the use of CBT screws could provide better stability (27). As the CBT screw trajectory was through cortical bone (higher-density bone), more reliable fixation was provided. Santoni et al. (12) found that CBT screws could provide up to a 30% increase in resistance to pull out compared with traditional pedicle screws, and 52% superior resistance in flexion and 35% in extension were also offered (28). Therefore, patients with low bone quality (e.g., patients with osteoporosis) were advised to receive CBT screws to decrease internal fixation failure.

Significant improvements in neurological symptoms were shown in patients with degenerative lumbar diseases by CBT screws in our study (based on JOA and ODI scores, *p* < 0.0001), and similar results were observed in several studies. Snyder et al. (15) adopted a study of 79 patients who suffered degenerative lumbar diseases and underwent surgeries with CBT screws; seventy patients (88.6%) improved without any complications. Dabbous et al. (29) found a clear improvement in ODI (59% vs. 34%) and a significant reduction in the use of analgesia in patients who were undergoing surgeries with CBT screws. Mori et al. (30) also reported that good leg pain relief was achieved in all patients with CBT screws, and JOA scores improved significantly (12 ± 4.9 vs. 28 ± 1.8, *p* < 0.001) at the final follow-up. CBT screws could provide perfect stability and bone fusion, and the starting point was at the crosshair of bisection of the pars interarticularis, which significantly decreased muscular manipulation and blood loss (31–33), which could explain the alleviation of neurological symptoms in patients.

We also assessed the HRQOL of patients based on the ODI score and MCID and found that elderly patients were more satisfied after surgery (Table 2). Several, previous studies demonstrated that the extent of recovery was negatively associated with age. Scheer et al. (34) reported that elderly patients (especially >65 years old) had higher SRS-22 scores than younger patients. Carreon et al. (35) also found that each 1-year increase in age was related to a 0.26-point decrease in the 2-year SF-36 after spine deformity surgery. Our previous study (36) also demonstrated that patients’ ages (e.g., those between the ages of 60–70) (odds ratio = 2.536, 95% confidence interval 1.214–5.300) were a predictive factor for a satisfactory recovery. Baseline disability and age are very closely related as previously described in the literature (37, 38). These findings indicated that patients with poor baseline conditions were inclined to achieve satisfaction. Our study also suggested similar results. We compared the severity of spondylolisthesis before surgery, and the extent of spondylolisthesis in elderly patients was more severe (Group A versus Group B (preoperative: *p* = 0.023, mGrade: sGrade: OR=0.200). We postulated that elder patients presented with more disabilities and complications than younger patients; thus, elder patients had lower prospects and a more acute perception of the surgical effect, which meant that a slight improvement equaled more satisfaction in elder patients.

Posterior spinal fixation is performed with pedicle screws for various spinal conditions requiring stabilization, such as trauma, deformity, tumor, and/or spondylolisthesis. However, loosening of the screw-bone interface, known as “screw loosening,” may result in pain and loss of correction. The risk of screw loosening is particularly high in patients with osteoporosis because an inferior reduction of intravertebral screws presents a great challenge for spine surgeons. Jose et al. (39) believed that CBT screws can improve the fixation strength of patients with osteoporosis. Thus, we suggested that the indications of CBT screw included severe osteoporosis, pedicle dysplasia, thin pedicle, and repeated screw placement caused pedicle rupture.

In our study, we conducted a morphometric measurement of the cortical screw trajectory using 100 consecutive CT scans of the lumbar spine of patients with degenerative diseases. We also provided the data separately, based on age and sex. The starting point was at the crosshair of the center of the superior articular process and 1 mm inferior to the borderline of the transverse process, which Ivanov et al. (40) demonstrated as the thickest part of the pars interarticularis. The position of the starting point also avoided touching the nerve root that passed beneath the lumbar vertebrae. We also compared the trajectory data between patients who were ≥60 and ≤45 years old, and in general, no significant differences were observed. However, the trajectory data of males were larger than those of females. These findings demonstrated that no impact of degenerative conditions existed and that sexual factors were greatly influenced. Zhang et al. (41) found that the distances from the starting point to the inferior border of the inferior articular process at the upper level varied in patients aged ≥ or <60 years old, which indicated that studies with larger samples are needed. The cephalad angle ranged from 23.5° to 28.6° in Group I, 24.7° to 26.6° in Group II, 25.0° to 28.2° in Group III, and 24.7° to 27.9° in Group IV, which were generally similar to the ranges reported by Zhang et al. (41) (Chinese) and Matsukawa et al. (42) (written in Japanese) in Asia. The lateral angle was also similar to that reported by Matsukawa et al. (42). We found that the maximum transverse length ranged from 35.5 to 39.4 mm in Group I, 35.7 to 40.1 mm in Group II, 35.3 to 37.1 mm in Group III, and 33.4 to 39.0 mm in Group IV, which was similar to those reported by Matsukawa et al. (42) (from L1 to L5: 36.8 ± 3.2, 38.2 ± 3.0, 39.3 ± 3.3, 39.8 ± 3.5, and 38.3 ± 3.9 mm).

Our study elucidated satisfactory outcomes for using CBT screws in degenerative surgeries in combination with reliable and safe trajectory data. Previous studies have also suggested several drawbacks that should be considered, such as the starting point or pedicle fractures with increased screw diameter (43) and upper nerve root injury by the correct depth of screw penetration (42). Matsukawa et al. (13) also indicated that cranial facet joint violations with CBT screws occurred in 11.8% (48/404) of patients, especially in patients aged >70 years, those with adjacent facet joint degeneration, and those with vertebral slip >10%. Glennie et al. (34) also demonstrated that 2/8 of patients with CBT screws needed revision because of pseudarthrosis and caudal adjacent segment failure (with screw loosening). Glennie et al. believed that the resultant increase in micromotion might lead to early cyclic loading failure; thus, the stiffness of the construct was essential, and an interbody device was needed when using CBT screws. However, we did not observe any postoperative complications, which might be attributed to the thorough understanding of the anatomy and accurate surgical procedures of the chief doctor. Overall, however, further, follow-up is needed.

The present study is subject to several limitations. First, it was a case series and did not compare the clinical outcome of CBT screws with traditional fixation that had limited power to evaluate the efficacy of using CBT screws in treating degenerative scoliosis. Second, this was a single-center study, meaning it limited sample size even though it provided satisfactory reliability and validity. Furthermore, the morphometric measurements were conducted based on CT, which were performed in the prone position, which might have influenced the accuracy of measurement. Additionally, the assessment of osteoporosis was not included in our study because of high-dose radiation, which might influence the accuracy of the results.

## Conclusion

This study resulted in detailed trajectory data based on sex and age separately, which might provide researchers with clinical guidance. We performed a case series to evaluate the efficacy of surgery with CBT screws and found that CBT screws could significantly improve degenerative lumbar diseases in patients, especially elderly patients.

## Data Availability

The raw data supporting the conclusions of this article will be made available by the authors, without undue reservation.
